# An empirically derived method for measuring human gut microbiome alpha diversity: Demonstrated utility in predicting health-related outcomes among a human clinical sample

**DOI:** 10.1371/journal.pone.0229204

**Published:** 2020-03-02

**Authors:** Sarah L. Hagerty, Kent E. Hutchison, Christopher A. Lowry, Angela D. Bryan

**Affiliations:** 1 Department of Psychology and Neuroscience, University of Colorado Boulder, Boulder, CO, United States of America; 2 Veterans Affairs Palo Alto Health Care System, Palo Alto, CA, United States of America; 3 Department of Integrative Physiology, University of Colorado Boulder, Boulder, CO, United States of America; 4 Center for Neuroscience, University of Colorado Boulder, Boulder, CO, United States of America; 5 Center for Microbial Exploration, University of Colorado Boulder, Boulder, CO, United States of America; University of Hawai'i at Manoa College of Tropical Agriculture and Human Resources, UNITED STATES

## Abstract

The human gut microbiome has emerged as a potential key factor involved in the manifestation of physical and mental health. Despite an explosion of cross-disciplinary interest in researching the gut microbiome, there remains to be a gold-standard method for operationalizing gut microbiome alpha diversity. Given researchers’ interest in examining the relationships among gut microbiome alpha diversity and health-related outcomes of interest, a way of operationalizing the microbiome that yields a numeric value, which could be used in common statistical approaches, is needed. Thus, the current study aims to provide methodological guidance for how to operationalize microbiome alpha diversity. Findings suggest that alpha diversity of the human gut microbiome is comprised of two sub-constructs (richness and evenness), and we propose a step-by-step method of creating alpha diversity composite measures based on this key insight. Finally, we demonstrate that our empirically derived richness and evenness composite measures are significantly associated with health-related variables of interest (alcohol use, symptoms of depression) among a human clinical sample.

## Introduction

Over the past two decades, the gut microbiome has received extensive attention from research communities due to its apparent involvement in a host of medical and psychiatric disorders [1]. Some researchers have begun to think of it akin to another body organ, which interacts with the host to promote health and, in some instances, initiate disease. As a point of nomenclature, the microbial species that inhabit the gastrointestinal tract are known as *gut microbiota*, while the genomes of the microbiota are known as the *gut microbiome*. A common, measurable outcome used in microbiome research is the diversity of microbes within a given body habitat, which has been defined as the number, abundance, and distribution of organism types [2]. Relatedly, the microbiome can characterize the abundance and diversity of species of gut microbiota [3]. Methodologically, human feces are representative of these interpersonal differences [4], making fecal samples a viable sample to collect for such investigations.

Current objectives of microbiome research include understanding the mechanistic role of microbial diversity in disease etiology and human health. Towards these ends, researchers have outlined factors that drive variation in microbiome environments. Among these factors are age, such that microbiome environments are volatile in human infancy and become more stable after the first three years of the lifespan [5]. Furthermore, genetics, environment, and lifestyle factors (e.g., diet, alcohol consumption) appear to influence the human gut microbiome [5,6]. For example, alcohol consumption is one such lifestyle factor that seems to be associated with composition of the human gut microbiome and gut health more broadly. Specifically, alcohol consumption is associated with greater intestinal permeability [7]. In the context of alcohol use disorder, intestinal permeability has been linked to increased levels of proinflammatory signaling molecules [8] and gut microbiome dysbiosis (an imbalance of microbial species within the gut) [7]. Though the causal relationship between gut microbiome composition and inflammation are still being characterized, these initial studies support a theoretical model in which gut microbiome composition underlies the manifestation of disease states via interactions with other biological systems, such as the immune system. Alcohol use disorder is just one of many areas where the microbiome is emerging as an important biological mechanism that could help explain the causes and consequences of various disease states.

A better understanding of the human gut microbiome, and the factors that drive variability with respect to microbiome composition, could lead to important insights in the areas of mental and physical health. A compelling body of research suggests that the gut-brain axis, which refers to the bidirectional influence between the gastrointestinal tract and the brain [9], might underlie the manifestation of disease and health dysfunction [10]. The composition of the gut microbiome plays a fundamental role in the gut-brain axis [11]; therefore, studying gut microbiome composition is of principle importance in order to better comprehend the etiology and consequences of physical and mental health dysfunction and refine maximally effective treatments.

Despite the relevance of gut microbiome composition to human health, there is not yet a gold standard, empirically based operationalization of gut microbiome alpha diversity, which captures the diversity of species within a given sample [12–14]. In the literature to date, many existing studies relating alpha diversity to human outcomes have selected individual alpha diversity indices to use in statistical models. For example, Vogt and colleagues [15] separately tested whether two alpha diversity metrics, abundance-based coverage estimator (Ace) and Chao1, were associated with diagnostic (i.e., presence of Alzheimer’s disease) and biological (i.e., cerebrospinal spinal fluid) outcomes. Furthermore, a recently published study tested whether three alpha diversity metrics (observed operational taxonomic units (OTUs), Shannon, and Chao1) were associated with attention-deficit/hyperactivity disorder (ADHD) [16]. Additionally, Jiang and colleagues [17] found that Shannon diversity differed among patients who met criteria for major depressive disorder and healthy controls. As exemplified by these studies, a common strategy employed in the existing literature is to select *a priori* individual alpha diversity indices to represent gut microbiome diversity. An examination of the literature [18], including the mathematical expressions that are used to derive these alpha diversity measures [19], suggest that the construct of alpha diversity comprises both *richness* (i.e., how many different kinds of species are present within a given community) as well as *evenness* (i.e., the extent to which species within a community are held in even abundance with one another). Importantly, no published study to date has empirically tested whether these richness/evenness sub-constructs of alpha diversity emerge within human samples.

There exist a number of documented alpha diversity indices, thus providing an opportunity to empirically derive a composite measure based on multiple indices of alpha diversity. One commonly used method for building composite measures is to run an exploratory factor analysis and create composite variables based on factor loadings [20]. One particular study investigated the statistical benefits of using composite measures as compared to individual indicator variables. The results of this study suggest that model fit is improved when using composites compared to treating all indicator variables individually, which is especially true at smaller sample sizes [20]. Another study evaluated the reliability of composite measures of pain compared to individual pain measures. Findings suggest that the composite pain measures demonstrated superior reliability (as defined by stability over time throughout the follow-up period) compared to the individual measures [21]. These findings are consistent with what is known about the statistical properties of multi-item scales. That is, writings on quantitative methodology argue that composite measures reduce measurement error, increase reliability, and result in improved normality [22] thereby improving the overall robustness of the measure.

Therefore, the aims of the current study were twofold: 1) to empirically derive a robust and reliable measure of gut microbiome alpha diversity; and 2) to demonstrate the utility of the derived gut microbiome metric with regards to statistically predicting measures of human anthropometrics (i.e., age), behavior (alcohol use and fruit/vegetable consumption), and health characteristics (depressive symptoms).

## Materials and methods

### Study design

The present set of analyses employed data from a larger parent study, which tested the effectiveness of behavioral interventions among a sample of heavy alcohol drinkers who reported wanting to quit or reduce their drinking. The parent study was approved by the University of Colorado Boulder Institutional Review Board, and all participants provided written informed consent at the beginning of the baseline appointment. Only elements of the parent study that are relevant to the current set of analyses will be outlined here. The current investigation includes data from the baseline assessment prior to patients engaging in the intervention. At the time of the baseline assessment, patients completed self-report and researcher-administered assessments. Additionally, participants were provided a stool sample collection kit and were instructed to return a viable sample to the lab at the time of their first intervention session.

### Participant selection and eligibility

Advertisements in mass media outlets (e.g., newspaper, radio), social media (e.g., Facebook), and direct mailings targeting the greater Front Range metropolitan area, which includes Boulder County, Denver, and suburbs of Denver, were the primary sources of participants. Participants who were deemed eligible following a phone screening procedure scheduled their baseline appointment. For the current set of analyses, participants included a final sample of 64 individuals who were selected for inclusion according to the following criteria: 1) must have been between the ages of 21 and 60 and provided informed consent; 2) had a primary DSM-5 diagnosis of alcohol use disorder; 3) were within 10 days of last drink at the time of the baseline assessment; 4) had been drinking heavily (criteria dependent upon individual’s age, gender, and BMI) for a consistent period of time; 5) had a breath alcohol level of 0 at screening (to sign consent form); 6) were not currently taking any medications for the treatment of psychiatric disorders, including substance use disorders, mood disorders, and psychosis; 7) female participants were not pregnant, as indicated by a pregnancy test administered at baseline; 8) tested negatively for sedatives, opiates, cocaine, or amphetamine on drug screen at baseline; 9) did not meet criteria for a psychotic disorder or bipolar disorder; 10) had a Clinical Institute Withdrawal Assessment (CIWA) score less than 8 (indicating no need for medical detox); 11) had expressed a desire during their initial screen to reduce the number of drinks they regularly consume; 12) had their baseline stool sample sequenced for analysis of microbiome composition by the initiation of data analysis.

### Sample characteristics

All 64 participants completed each of the study procedures relevant to the current set of analyses outlined below. Behavioral and biological data for these 64 individuals were collected between September 2016 and December 2018. A summary of relevant sample characteristics at baseline can be found in Table 1. The sample includes middle-aged adults (mean = 46.3 years), and is broadly representative of the Boulder/Denver metropolitan areas with regards to race (92.2% Caucasian). On average, participants reported moderate depression based on responses to the Beck Depression Inventory (BDI; mean = 12.5; [21]). A validated self-report measure of fruit and vegetable consumption [23] suggested that, at baseline, participants consumed 2.6 daily servings of fruits and vegetables on average.

**Table 1:** Characteristics of sample at baseline.

Measure	Mean	Standard Deviation
Age	46.34	9.30
AUDIT	19.39	8.07
Drinking Days/30 Days	23.27	5.47
Total Drinks/30 Days	103.82	62.26
Drinks/Drinking Day	4.47	2.37
BDI	12.45	7.54
Fruit and Vegetable Servings	2.57	2.00

AUDIT = Alcohol Use Disorders Identification Test; Drinking Days/30 = number of days over the past month alcohol was consumed as reported on the 30-day Timeline Follow Back (TLFB); Total Drinks/30 Days = total number of drinks reported on the TLFB over the preceding 30 days; Drinks/Drinking Day = average number of drinks per days alcohol was consumed as reported on the TLFB; BDI = Beck Depression Inventory; Fruit and Vegetable Servings = average number of fruit and vegetable servings consumed per day over the month preceding the baseline session according to Subar et al. (2001).

Given the nature of the parent study, particular emphasis was placed on characterizing the sample with regards to alcohol use. At baseline, participants reported hazardous or harmful alcohol use indicative of dependence based on AUDIT score (mean = 19.4; [24]). Generally, an AUDIT score of eight or above is considered harmful use, while an AUDIT score of 11 or more is indicative of alcohol dependence [25]. The TLFB [26] was employed to quantify alcohol and other substance use across the preceding 30 days. At baseline, participants reported drinking approximately 23 days in the preceding month, and an average of 4.5 drinks on each drinking day (see Table 1).

### Study procedures

Relevant to the current set of analyses, after arriving at the lab for their baseline appointment, qualified participants: 1) completed an informed consent form; 2) filled out baseline self-report questionnaires; 3) completed researcher-administered assessments; and 4) were also given a gut microbiome sample collection kit at the end of the session to be returned during their first scheduled intervention appointment.

The self-report measures that were administered included a number of individual difference measures, such as a demographics questionnaire that was used to collect information on participants’ race, age, and sex. Participants also completed the Alcohol Use Disorders Identification Test (AUDIT) [24], which was used to assess symptoms of alcohol dependence. In order to assess the quality of participants’ diet, participants completed The Fruits and Vegetable Screener [23], a 10-item measure that was developed by the National Cancer Institute to assess how many times in the previous month participants consumed different types of fruits and vegetables, including portion size questions for every food item. This measure was shown to be a useful estimate of median intakes of fruit and vegetable servings in U.S. populations, and has been recognized as a proxy measure for quality of diet. In addition to the self-report measures described above, participants worked with a research assistant to complete a TLFB assessment. The TLFB is a calendar-based assessment of daily alcohol and drug use [27] that has been shown to have good psychometric characteristics among a variety of drinker groups and can generate variables that provide a wide range of information about an individual's drinking (e.g., pattern, variability, and magnitude of drinking).

### Gut microbiome sample collection

As mentioned, participants were provided with a fecal sample collection kit during the baseline assessment, and they were instructed to return the sample collection kit to the lab at the time of their first therapy appointment. Participants provided a stool sample by using a BD BBL CultureSwab Sterile, Media-free Swab (Cat. No. 220145, Becton, Dickinson and Company, Sparks, MD, USA) and a piece of used toilet paper. Participants were instructed to collect a sample within a day of providing it to the study staff. The sample was stored it at –80°C until molecular processing.

### Molecular processing

DNA was extracted using the PowerSoil DNA extraction kit (Cat No. 12888–100 & 12955–4, MoBio Laboratories, Carlsbad, CA, USA) according to the manufacturer’s instructions. Marker genes in isolated DNA were PCR-amplified using HotStarTaq Master Mix (Cat No. 203433, Qiagen, Valencia, CA, USA) and 515 F (5’-GTGCCAGCMGCCGCGGTAA-3’) 806 R (5’-GGACTACHVGGGTWTCTAAT-3’) primer pair (Integrated DNA Technologies, Coralville, IA, USA) targeting the V4 hypervariable region of the 16S rRNA gene modified with a unique 12-base sequence identifier for each sample and the Illumina adapter as previously described in Caporaso et al., 2012 [28]. The thermocycling program consisted of an initial step at 94°C for 3 min followed by 35 cycles (94°C for 45 sec, 55°C for 1 min, and 72°C for 1.5 min), and a final extension at 72°C for 10 min. PCR reactions were run in duplicate and the products from the duplicate reactions were pooled and visualized on an agarose gel to ensure successful amplification. PCR products were cleaned and normalized using a SequalPrep Normalization Kit (Cat. No. A1051001, ThermoFisher, Waltham, MA, USA) following manufacturer’s instructions. The normalized amplicon pool was sequenced on an Illumina MiSeq run by using V3 chemistry and 600 cycle, 2 x 300-bp paired-end sequencing. All sequencing and library preparation were conducted at the University of Colorado Boulder BioFrontiers Next-Gen Sequencing core facility, https://bficores.colorado.edu/sequencing-lab.

### Microbial data processing and preliminary analysis

After the raw sequencing files were generated, data were processed using Quantitative Insights Into Microbial Ecology (QIIME 2 version 2018.11) open source software [29]. A metadata file for each run was validated using the Keemei Google Sheets add-on [30]. Raw sequencing files were demultiplexed and subsequently analyzed for quality by using the QIIME visualizer to examine the interactive quality plots. De-noising parameters (i.e., trimming sequence read lengths) were specified for each run according to the following: 1) demonstrated a minimum of 150 nucleotides in both forward and reverse reads; and 2) maintained quality scores at or above 30. Rarefaction was conducted according to [31]. A tree was generated for downstream phylogenic diversity analyses by aligning sequences, generating an ‘unrooted’ tree, and then ‘rooting’ the tree. Taxonomy was assigned using the Ribosomal Database Project classifier [32] trained on the Greengenes 13_8 16S rRNA gene database [33]. Several alpha diversity metrics were calculated in QIIME 2 (see Table 2 for full list of alpha diversity measures that were calculated).

**Table 2:** Alpha diversity measures and descriptions.

Alpha Diversity Measure	Description	Citations
Ace	An estimate of species richness using a correction factor	[18,34]
Observed OTUs	The number of different OTUs per sample	[16]
Chao1	A measure of species richness, which gives more weight to rare species	[34]
Margalef	Measure of species richness in a given area or community	[35,36]
Fisher alpha	Relationship between the number of species and the relative abundance of each species; referred to as a measure of richness	[37,38]
Faith pd	Sum of OTU branch lengths; takes into account phylogenic distance between OTUs; the greater the number of unique OTUs, the higher this index will be	[18,39]
Brillouin d	Measures the diversity of the species present	[18]
Shannon	Calculation of richness and evenness using a natural logarithm; accounts for both relative abundance and evenness of the taxa present; referred to as an attempt for one metric to capture both richness and relative evenness	[18,40]
Enspie (ENS)	Measure of the number of equally abundant species; if the relative abundance of all species in a community were exactly identical, ENS would simply be the total number of species in that community	[41]
Menhinick	The ratio of the number of taxa to the square root of the sample size; referenced as "species richness"	[18,35]
Mcintosh e	A measure of how evenly/unevenly abundant taxa are in the sample	[18,42]
Simpson	Measure of relative abundance of the different species making up the sample richness; referred to as an attempt for one metric to capture both richness and relative abundance	[40,43]
Berger Parker d	A measure of relative richness of the abundant species; higher values indicate relative abundance disturbance	[18,44,45]
Strong	Assesses species relative abundance unevenness/dominance concentration	[46]
Simpson e	A measure of evenness based on the number of organisms and number of species	[43]
Pielou e	Measure of relative evenness of species richness	[12,42,47]
Heip e	A measure of evenness	[18,42]
Lladser pe	A measure of how much of the environment contains unsampled taxa	[18,48]

### Statistical analysis

#### Aim 1: Deriving a composite gut microbiome diversity metric

*Selecting viable alpha diversity metrics*. Alpha diversity of species within the gut microbiome is a common outcome of interest that is examined in microbiome research. Analysis of the composition of the microbial community involves sequencing of the microbial community DNA, and matching the sequences to known functional genes [5]. Aim 1 of the current study involved empirically deriving a composite numerical metric that represents microbiome alpha diversity, which could be used as a predictor or outcome of interest in common statistical models. As discussed above, a composite measure comprised of alpha diversity metrics could be a useful way to operationalize gut microbiome alpha diversity.

Alpha diversity metrics from the baseline microbiome samples output from QIIME 2 [29] were evaluated for inclusion in an exploratory factor analysis (EFA). Briefly, EFA is a statistical technique where the input is a correlation matrix of observed variables and the output is a summary of how each observed variable maps onto latent, unobserved “factors.” For further comprehensive descriptions of factor analytic methods see [49–52]. Because the number of latent factors is typically far fewer than the number of observed input variables, factor analysis is often considered to be a useful data reduction technique. Importantly, there are other statistical techniques that allow researchers to explore the underlying relationships among variables, including principal component analysis (PCA). Mathematically, the difference between PCA and EFA concerns the variance that is analyzed among the observed variables, such that all the variance in the observed variables is analyzed in PCA and only the shared variance among the observed variables is analyzed in EFA. Theoretically, the difference between PCA and EFA lies in the assumptions made about the nature of the relationship between the set of observed variables and the latent, unmeasured factor(s)/component(s). That is, factor analysis supposes that the measured indicator variables are caused by latent, unmeasured forces (i.e., the latent factors). As articulated in a useful chapter by [53], factor analysis explores the underlying processes that could have produced correlations among a given set of variables. In contrast, PCA supposes that the individual indicator variables cause the component(s). As such, components derived from a PCA analysis can be thought of simply as aggregates of the set of indicator variables. In other words, variables that comprise a component are merely empirically associated and are not necessarily thought to reflect an underlying process.

In the case of the present analysis, our choice to use EFA was based on our theoretical understanding of the alpha diversity construct. That is, using the EFA approach is consistent with our implicit hypothesis that an underlying process(es) (e.g., biological, environmental) cause(s) certain indicator variables to be correlated with one another. Therefore, our choice to use EFA over PCA was made due to the directionality of our theory-based understanding of the relationship among the alpha diversity construct and measurable indictor variables thought to measure alpha diversity. In summary, the choice to use EFA instead of PCA was an a priori, theory-driven decision. However, it is important to note that there is an ongoing debate among statistical theorists and researchers regarding when to use EFA and PCA [54]. When electing to use an EFA approach, we also had to determine whether EFA or confirmatory factor analysis (CFA) was most appropriate. While some have noted that alpha diversity of the gut microbiome may be comprised of richness and evenness processes (i.e., two proposed factors), we believe that it is still prudent to continue to explore whether this hypothesized structure of underlying processes is supported by empirical data in an exploratory fashion. Therefore, we elected to specify an EFA as opposed to testing the two-factor CFA model.

Prior to inclusion in the EFA, some alpha diversity metrics were excluded on the basis of being highly correlated with other alpha diversity metrics. Rather than selecting an ad-hoc cutoff for correlation strength, our strategy was to include the greatest number of observed variables as possible while simultaneously attempting to remove redundancy. Towards these ends, we evaluated the pattern of correlations between observed variables, and retained metrics that seemed to represent unique aspects of alpha diversity based on the correlation matrix. For example, ace, observed otus, chao1, margalef, and fisher alpha are correlated with one another at *r* >.98, therefore, we elected to retain one representative metric from that cluster of four metrics (fisher alpha). Some instances of high correlations were addressed by removing metrics due to elevated skewness and kurtosis. For example, removing mcintosh e because of elevated skewness and kurtosis took care of the high correlation between mcintosh e and Shannon (*r* = -.94) and between mcintosh e and simpson (*r* = -.96).

Next, alpha diversity metrics were evaluated for inclusion in the EFA based on whether there was an indication that the variable did not meet the assumption of normality. Specifically, some alpha diversity metrics were excluded from the EFA if they demonstrated elevated skewness and/or kurtosis, which are numerical indicators of whether a given variable is normally distributed. We chose to exclude indicator variables on the basis of their skewness and/or kurtosis because non-normality can affect the integrity of some statistical tests, including factor analysis. Many statistical tests are based on the normality assumption [53]. Skewness and kurtosis are measures of normality such that a skewness value different from 0 indicates that the distribution deviates from symmetry, and a kurtosis value different from 0 indicates that the distribution deviates from normality in tail mass and shoulder [55]. Cain and colleagues (2016) evaluated the impact of skewness and kurtosis in the context of several statistical tests, including CFA. Specifically, authors simulated data with a known factor structure, manipulated the skewness and kurtosis of the data, and evaluated the Type I error rate of rejecting the factor model using the chi-squared-goodness of fit test. Results suggest that elevated skewness and kurtosis result in Type I error rate of 30% in factor analysis [56]. Authors suggest that researchers should report skewness and kurtosis so that results can be evaluated in the context of the underlying variables meeting the assumptions of the normal distribution. Additionally, authors suggest that researchers should consider taking steps to modify their analyses to mitigate the impact of non-normally distributed variables on their analyses. Based on the methodological literature discussed above, we chose to eliminate indicator variables that demonstrated elevated skewness and kurtosis values. While some methodological research has highlighted the consequences of including variables with non-normal distributions in univariate and multivariate statistical analyses [56–58], there does not yet exist any concrete guidance in terms of numerical thresholds for indices that measure normality. Specifically, there is no agreed upon answer to the question: “how high of a kurtosis/skewness value is *too* high?” Therefore, we decided to eliminate indicator variables that demonstrated elevated skewness and kurtosis relative to the other indicator variables. See Table 3 for a summary of alpha diversity metrics that were evaluated, and reason for exclusion from the EFA (if applicable).

**Table 3:** Summary of alpha diversity metrics.

Alpha Diversity Measure	Included in EFA	Reason for exclusion
Ace	No	High inter-correlation with other indicators
Observed OTUs	No	High inter-correlation with other indicators
Chao1	No	High inter-correlation with other indicators
Margalef	No	High inter-correlation with other indicators
Fisher alpha	Yes	
Faith pd	Yes	
Brillouin d	No	High inter-correlation with other indicators
Shannon	Yes	
Enspie (ENS)	Yes	
Menhinick	Yes	
Mcintosh e	No	Elevated Skewness/Kurtosis
Simpson	No	Elevated Skewness/Kurtosis
Berger Parker d	No	Elevated Skewness/Kurtosis
Strong	Yes	
Simpson e	Yes	
Pielou e	No	Elevated Skewness/Kurtosis
Heip e	Yes	
Lladser pe	Yes	

**Fig 1:**
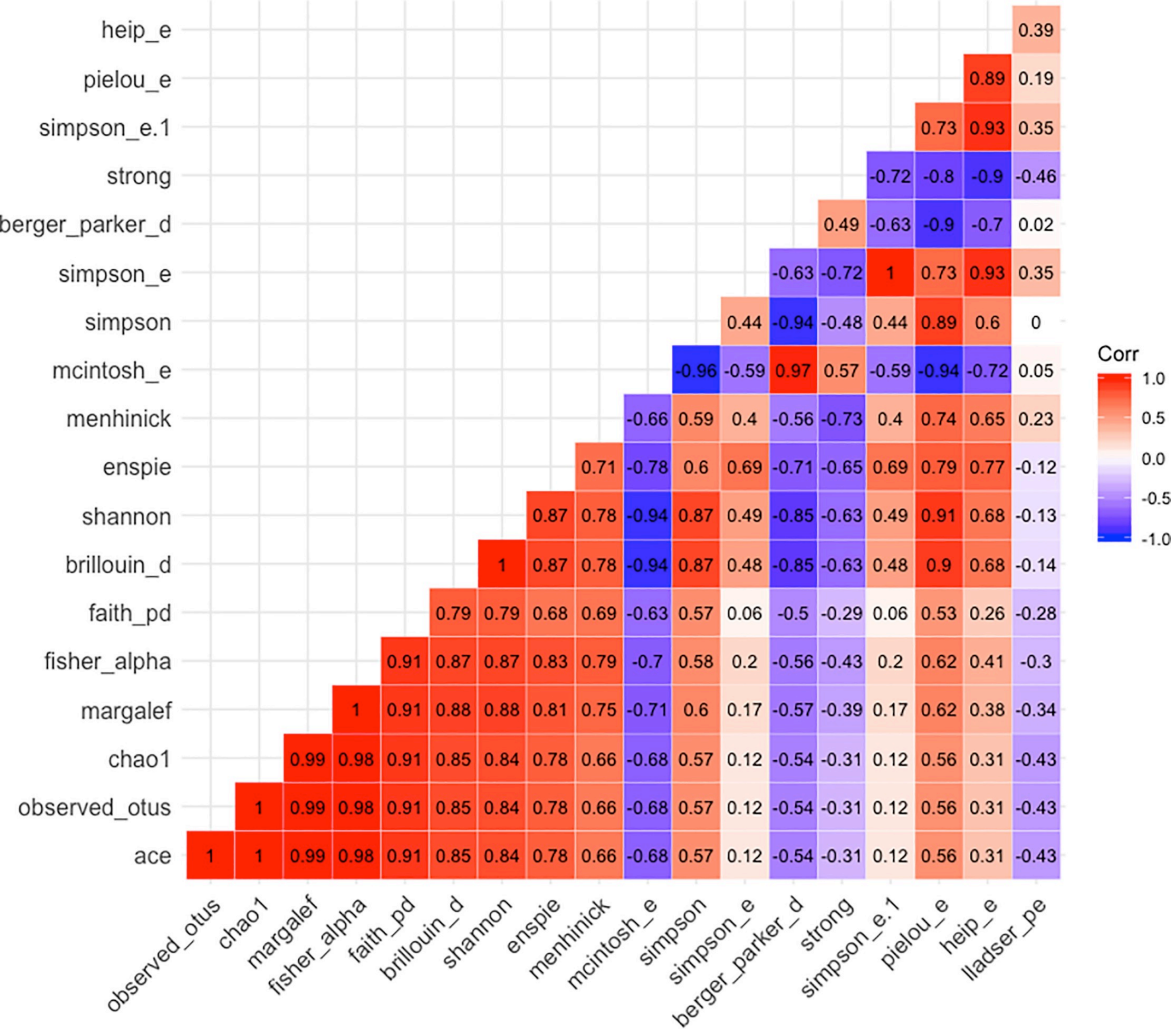
Correlations among alpha diversity indices. Correlations among all calculated alpha diversity indices are displayed. Positive correlations displayed in blue, and negative correlations displayed in red. Stronger correlations denoted by greater opaqueness.

OTUs = Operational taxonomic units; See Fig 1 for the correlation value between each pair of alpha diversity indices evaluated.

*Exploratory factor analysis*. The final set of nine viable alpha diversity metrics was advanced to the factor analysis stage. First, a scree plot was constructed in R version 3.5.3 in order to determine how many factors likely underlie the set of diversity indicators. After evaluating the scree plot and eigenvalues associated with each indicator variable, an exploratory factor analysis was specified with the baseline microbiome data in R [59]. Factor analytic approaches demonstrate considerable utility as a data reduction tool by reducing many variables down to fewer factors assuming a latent factor structure. Such approaches summarize the patterns of correlations in the observed correlation matrix to yield a more parsimonious and robust structure that is often more reliable than the original variables [53].

Specifically, all nine viable alpha diversity metrics were input into an EFA function specified with a varimax rotation and maximum likelihood factoring method. Based on the results from the EFA (see results section), a two-factor solution was adopted consistent with the theoretically relevant richness and evenness factors. Based on the pattern of factor loadings, searching the literature to derive a theoretical understanding of each alpha diversity measure, and examining the mathematical equations that are used to calculate each measure [19], the two factors were labeled “richness” and “evenness.” While we allowed the results of the EFA to guide our decision to retain two factors we used our knowledge of the literature to both characterize those two factors as “richness” and “evenness” and to guide our decisions in an iterative way throughout the model building process, which is considered to be an accepted practice in the context of EFA [53]. That is, when the scree plot suggested a two-factor solution, we hypothesized that the EFA results would support a “richness” and “evenness” factor structure based on our knowledge of the alpha diversity literature. Because we hypothesized that the two-factor solution may relate to “richness” and “evenness” factors, we chose to specify the model with a varimax rotation. Varimax, the most commonly used rotation, is a variance maximizing procedure that maximizes the variance of factor loadings (i.e., makes high loadings higher and low loadings lower) [53]. Varimax assumes, and mathematically encourages, an orthogonal factor structure [60,61]. Importantly, an orthogonal factor structure is consistent with the working theoretical understanding of how the richness and evenness sub-categorizations of alpha diversity measures relate to one another. That is, Mason and colleagues [62] argue that richness and evenness are orthogonal to one another (i.e., vary independently of one another). Thus, specifying the EFA with a varimax rotation is consistent with a theory-driven interpretation of the factor structure.

In order to derive composite scores for each factor, each alpha diversity metric was transformed into a z-score. This standardization step was crucial given that each alpha diversity metric is measured on a different scale. Individual alpha diversity metrics were reverse scored according to theory prior to being converted into a standardized scale. Next, alpha diversity *z*-scores were averaged together according to the factor structure. This process resulted in a “richness score” and an “evenness score” for each participant. These composite measures were then used in subsequent statistical models (see below).

#### Aim 2: Association between derived diversity metric and human variables of interest

Aim 2 of the study set out to assess the utility of the gut microbiome diversity metrics derived in Aim 1 in the context of statistically predicting human outcomes. Towards these ends, we set out to evaluate the association between gut microbiome alpha diversity, age, diet, depressive symptoms, and recent alcohol consumption. These four human outcome variables were selected due to their apparent association with gut microbiome composition and their broad relevance to human health [63,64].

Simple regression models tested the relationships between the microbiome richness and evenness composite measures and the four human health-related variables of interest. Diet was operationalized as the average number of fruits and vegetables consumed over the previous 30 days per the Fruits and Vegetable Screener [23]. Alcohol use was operationalized as the total number of standard alcoholic drinks consumed over the 30 days preceding the baseline appointment according to the TLFB assessment [27]. Participant age was based on their response on the demographics questionnaire. Finally, symptoms of depression was operationalized as participants’ scores on the BDI self-report measure [65].

First, baseline gut microbiota richness and evenness were regressed on baseline drinking behavior in separate models. Second, baseline gut microbiota richness and evenness were regressed on participant age in separate models. Third, baseline gut microbiota richness and evenness were regressed on average daily fruit and vegetable consumption, which is a proxy measure for quality of diet. Finally, baseline gut microbiota richness and evenness were regressed on BDI scores, which is a measure of participants’ current depressive symptoms.

In addition to testing the association between the human outcomes and the derived composite measures of microbiome diversity, simple regression models testing the association between alcohol use, age, diet, BDI scores and individual alpha diversity metrics were also specified. These models were identical to the models shown above, except instead of regressing the *composite* diversity measures on the human health and behavior variables of interest, *individual* alpha diversity measures (Faith pd, Strong) were regressed on each human outcome variable in separate models. This was meant to determine whether the composite measures derived in Aim 1 performed differently in their ability to statistically predict common human health and behavior variables of interest compared to individual diversity metrics (e.g., Faith pd). We chose to run regression models for only two individual measures of alpha diversity in an attempt to be sensitive to the issue of multiple comparisons because as the number of tests increase, so does the Type I error rate. Thus, we selected one “richness” measure and one “evenness” measure a priori in order to explore the question of whether the composite metrics provide predictive power over and above individual metrics of alpha diversity.

## Results

### Aim 1: Deriving a gut microbiome diversity metric

#### Selecting viable alpha diversity metrics

The full list of alpha diversity measures that were output by QIIME are shown and described in Table 2. The correlations among these alpha diversity measures are shown in Fig 1. Prior to inclusion in the EFA, some alpha diversity metrics were excluded on the basis of being highly correlated with other alpha diversity metrics. Rather than selecting an ad-hoc cutoff for correlation strength, our strategy was to include the greatest number of observed variables as possible while simultaneously attempting to remove redundancy. Towards these ends, we evaluated the pattern of correlations between observed variables, and retained metrics that seemed to represent unique aspects of alpha diversity based on the correlation matrix. For example, ace, observed otus, chao1, margalef, fisher alpha are correlated with one another at *r* >.98, therefore, we elected to retain one representative metric from that cluster of four metrics (fisher alpha). Some instances of high correlations were addressed by removing metrics due to elevated skewness and kurtosis. For example, removing mcintosh e because of elevated skewness and kurtosis took care of the high correlation between mcintosh e and Shannon (*r* = -.94) and between mcintosh e and simpson (*r* = -.96). See Fig 1 for the correlation value between each pair of alpha diversity indices evaluated.

In addition to examining correlations among the set of alpha diversity indicator variables, the distributions of the indicator variables were also examined in order to determine viability for inclusion in the EFA. Specifically, skewness and kurtosis were evaluated for each alpha diversity indicator variable. At this stage, 4 metrics were excluded on the basis of demonstrating elevated skewness/kurtosis, as evidenced by their skewness and kurtosis: Simpson (skewness = -5.03, kurtosis = 31.97), Pielou e (skewness = -1.84, kurtosis = 7.05), Mcintosh e (skewness = 2.71, kurtosis = 12.12), and Berger Parker d (skewness = 2.78, kurtosis = 11.54).

#### Exploratory factor analysis

**Fig 2:**
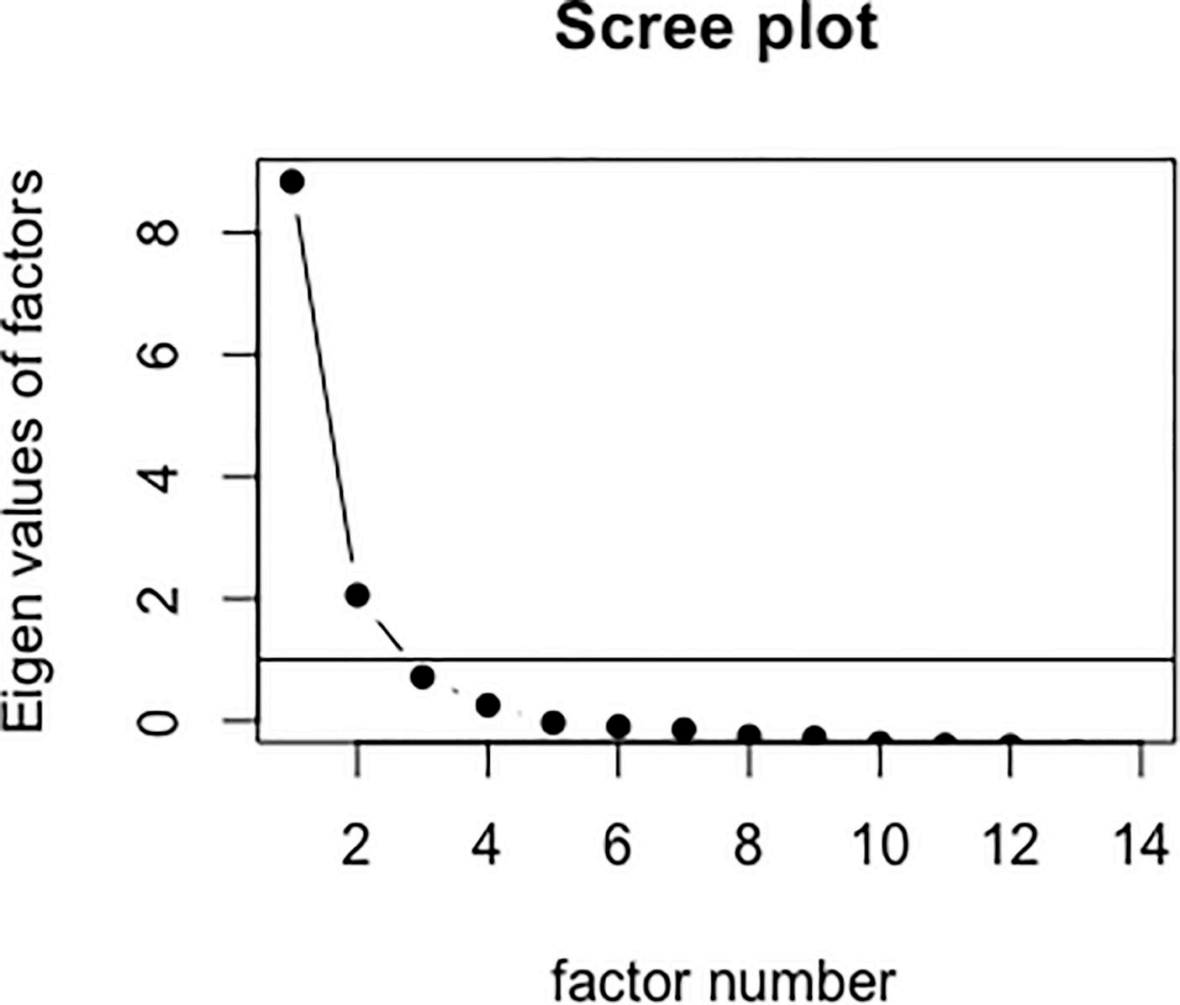
Scree plot of alpha diversity indices. Scree plot showing likely number of factors underlying the relationships among alpha diversity indices.

The final set of viable alpha diversity indicator variables were included in an EFA (see Table 3). In order to determine the likely number of factors among the sample of diversity indices, a scree plot was evaluated. Interpretation of the resulting scree plot (see Fig 2) suggests a two-factor solution. Specifically, two factors demonstrate eigenvalues greater than or equal to one, and common approaches to factor analysis suggest that eigenvalues greater than one are large enough to be retained [53]. An examination of the literature on diversity suggests that measures of alpha diversity capture two sub-constructs of diversity: richness and evenness [62,66–68]. Therefore, the scree plot, eigenvalues, and theory based on past literature all indicate a two-factor solution.

A summary of the exploratory factor analysis results is found in Table 4. As shown in Table 4, the factor loading pattern again supports a two-factor solution, as each alpha diversity indicator variable loads cleanly onto either of the two factors (i.e., factor loading >.5). Importantly, none of the alpha diversity indicator variables show a cross-loading pattern. That is, there are no indicator variables where the factor loading is >.5 for both factors (see Table 4). To ensure that these decisive loading patterns were consistent with theory, relevant literature/each measure’s mathematical expression [19] was consulted before categorizing a given alpha diversity measure as a measure of “richness” or “evenness.” For example, Faith pd loaded strongly onto the factor that has been labeled “richness” (.91 loading). Consultation of the literature confirms that it is appropriate to categorize Faith pd as a richness measure given that it is a measure of cumulative OTU branch lengths, and the greater the number of OTUs, the higher the Faith pd measure will be. This type of analysis was repeated for each alpha diversity measure.

**Table 4:** Results of the exploratory factor analysis.

Alpha Diversity Measure	“Richness” Factor Loading	“Evenness” Factor Loading	h2	Final Categorization
Fisher alpha	1.0		1.0	Richness
Faith pd	.91		.84	Richness
Shannon	.86		.89	Richness
Enspie (ENS)	.81		.90	Richness
Menhinick	.77		.74	Richness
Strong*		-.80	.81	Evenness
Simpson e		.93	.90	Evenness
Heip e		.92	1.0	Evenness
Lladser pe		.55	.40	Evenness

h2 = proportion of variance of each measure explained by the factors

* denotes measure that was “reverse coded” prior to *z*-score conversion on the basis of direction of factor loading and evaluation of relevant literature and mathematical expression; factor loadings > .5 shown.

Finally, in one case the factor loading was negative (i.e., Strong). In this case, an evaluation of the literature suggests that this negative factor loading is theoretically meaningful. The literature and evaluation of the mathematical equation associated with strong suggest that this measure does not measure evenness, but rather it measures *unevenness*, or species dominance. In other words, a higher score on the Strong measure suggests that the community being measured is more dominated by a fewer number of species [19]. Therefore, the negative factor loading in concert with an evaluation of the relevant literature and mathematical expression suggest that this measure should be reverse coded prior to including it in a composite measure. The Strong metric demonstrated observed values that ranged from 0–1. Therefore, in order to reverse code this measure, the inverse of the original measure was estimated by the following formula: 1-original measure. This reverse coding process was completed prior to Strong being converted to a *z*-score. The culmination of this set of analyses resulted in empirically derived and theoretically interpretable alpha diversity composite metrics (i.e., richness and evenness), which were then used to represent microbiome composition in statistical models.

### Aim 2: Association between derived diversity metric and human variables of interest

In order to assess the utility of the microbiome composition metrics derived in Aim 1, simple regression models tested the association between the richness and evenness composite measures and several variables of interest related to human health. Specifically, the associations between richness, evenness, age, recent alcohol consumption, symptoms of depression, and recent fruit and vegetable consumption were tested in separate models according to the models outlined in the statistical analysis section above. Results from each of these models are summarized in Table 5.

**Table 5:** Associations between microbiome composites and variables of interest.

	beta coefficient (*p*-value)			
Variable of Interest	Richness Composite	Evenness Composite	Faith pd	Strong
Drinks/30 days	**-.004 (.037)**	.00091 (.302)	**-.012 (.02)**	.0011 (.257)
Fruit/veggie servings	.008 (.913)	.045 (.13)	-.074 (.72)	.0038 (.287)
Age	-.014 (.251)	-.0025 (.677)	-.047 (.173)	.000065 (.92)
BDI	.0023 (.881)	**.021 (.0032)**	-.028 (.515)	-.0011 (.185)

BDI = Beck Depression Inventory; beta values and *p*-values correspond to the parameter estimate and *p*-value corresponding with the variable of interest in each row regression models specified in the statistical analysis section (where richness/evenness composites were regressed on each variable of interest in separate models); statistically significant parameter estimates and corresponding *p*-values at an α < .05 two-tailed threshold are shown in boldface type.

Given the aims of the current investigation, we were not only concerned with whether the composite measures of microbiome composition were significantly associated with the variables of interest. We were interested in what the pattern of results suggests regarding the utility of the derived microbiome composite measures. Importantly, the pattern of results lends support to what we determined in Aim 1: richness and evenness are two subcomponents of microbiome composition. In fact, richness and evenness seem to relate differently to human behavior. That is, richness, but not evenness, of species within the human gut microbiome was associated with recent alcohol consumption (*b* = -.004, *p* = .037, 95% CI: -0.0074, -0.00024) such that greater alcohol consumption is associated with less richness of species within the microbiome (as expected based on our working theoretical model). Additionally, richness and evenness of the gut microbiome appear to relate differently to current depressive symptoms. That is, evenness, but not richness, is significantly associated with participants’ scores on the BDI (*b* = .021, *p* = .0032, 95% CI: 0.0075, 0.035), such that greater depressive symptoms is associated with less evenness of microbial species. Though only two of the human variables of interested demonstrated differing relationships with richness and evenness based on meeting significance thresholds (*p* < .05), the pattern of results more generally (e.g., directionality and magnitude of parameter estimates) suggests that richness and evenness might differ with regard to their associations with human behavior (e.g., diet, alcohol consumption) and human characteristics (e.g., age, mental health).

Also of note is the finding that the gut microbiome composite measures detected significant associations when individual alpha diversity measures did not. Specifically, model results suggest that evenness of microbial species, operationalized by the evenness composite, is significantly associated with symptoms of depression. However, this effect was not detected when microbiome evenness was operationalized by the Strong metric (an individual alpha diversity metric thought to capture (un)evenness of microbial species). This suggests that the microbiome composite measures may be a more robust and powerful way to capture composition of the gut microbiome.

#### The impact of analytic decision making on results

Throughout the analytic process, decisions were made that could have impacted the results. Specifically, we made the decision to exclude some observed alpha diversity metrics from the EFA if a given metric demonstrated elevated skewness/kurtosis or if a given metric was redundant (i.e., highly correlated) with other alpha diversity metrics. In doing so, we intended to include a representative set of alpha diversity indices that contributed unique information and did not violate the assumption of normality. Though the intention behind these decisions was to enhance the robustness and integrity of the analysis, we acknowledge that these decisions could have impacted the results. In an effort to determine the possible impact of these decisions, we re-ran the scree plot and EFA including all alpha diversity metrics.

Consistent with the primary results, the scree plot in which all alpha diversity indices were included again indicated a two-factor solution. Thus, an EFA was specified with two factors. The results of this model are summarized in Table 6. The results are broadly consistent with the EFA results from the model that included the more exclusive list of alpha diversity indices. That is, the factor loadings support a two-factor solution. Each of the alpha diversity indices that was included in the more exclusive model demonstrates consistency between the two models in terms of the pattern of factor loading. For example, Shannon was loaded strongly onto the “richness” factor in the exclusive EFA (factor loading = .86), and Shannon loaded strongly onto the “richness” factor in the all-inclusive model (factor loading = .81). Interestingly, several alpha diversity metrics demonstrate a cross-loading pattern (the metric loads > .5 onto both the “richness” factor and the “evenness” factor) in the context of the model that includes the full set of alpha diversity metrics. Despite these cross-loadings, the pattern of factor loadings suggests that one factor represents “richness” and the other factor represents “evenness.” Given that the interpretation of results was consistent across the two models, it is reasonable to suggest that the decisions made regarding which factors to ultimately include in the EFA did not influence the results in a way that impacts the broad conclusions drawn.

**Table 6:** Results of the exploratory factor analysis with all observed alpha diversity metrics.

Alpha Diversity Measure	“Richness” Factor Loading	“Evenness” Factor Loading	h2
Ace	.99		.99
Observed otus	.99		.99
Chao1	.99		.99
Margalef	.98		.98
Fisher alpha	.96		.96
Faith pd	.91		.83
Brillouin d	.82	.56	.98
Shannon	.81	.57	.98
Enspie (ENS)	.68	.61	.84
Menhinick	.61	.52	.64
Mcintoch e	-.65	-.67	.86
Simpson	.57	.58	.65
Simpson e		.92	.85
Berger parker d	-.51	-.68	.73
Strong*		-.81	.70
Pielou e		.85	.96
Heip e		.98	.99
Lladser pe			.40

h2 = proportion of variance of each measure explained by the factors; factor loadings > .5 shown

## Discussion

### Summary of results

The current study aimed to make a novel methodological contribution to the literature by developing an empirically-derived composite metric(s) that captures gut microbiome alpha diversity, which could be used in common statistical modeling approaches (Aim 1). Alpha diversity emerged as a relevant construct due to the fact that alpha diversity broadly captures within-participant microbial diversity. Additionally, several alpha diversity metrics have been developed and documented [19], thus providing an opportunity to derive a robust composite measure from a host of existing metrics.

Results provide key insights into the broad construct of microbial diversity, and offer a novel method for operationalizing gut microbiome diversity. Results of the EFA empirically confirm what has been noted in the literature. That is, existing alpha diversity metrics capture two facets of microbial composition: “richness” and “evenness.” The current study provides one possible method by which to calculate these composite measures. The method suggested here is to reverse code metrics as needed, convert all metrics to standardized *z*-scores, and average together metrics that are characterized as “evenness” metrics and average together metrics that are characterized as “richness” metrics.

Results from Aim 2 of the current investigation demonstrate the utility of this methodological approach with regard to investigating the relationship between microbiome composition and variables related to human health. Results suggest that the microbiome composition composite metrics that were derived using the method described in the manuscript demonstrate significant associations with key variables of interest. Specifically, in this sample, microbiome richness was significantly associated with recent alcohol consumption, and microbiome evenness was significantly associated with self-reported symptoms of depression.

### Methodological considerations

The current study employs samples from human fecal samples, which were sequenced in order to extract gut microbiome data. The sample is a methodological strength and enhances the novelty of the current investigation for two main reasons. First, the sample is a clinical sample comprised of participants who meet criteria for a common health condition (alcohol use disorder), thus providing an opportunity to examine the relationship between the gut microbiome and variables related to human health using a clinical sample of treatment-seeking individuals. Second, the current study employed one of the largest sample sizes to date (*N* = 64) compared to existing studies on the human gut microbiome and alcohol use. For example, one study that investigated the effect of diet on the human gut microbiome was published in the journal *Nature* in 2014 with a sample size of *N* = 10 [6]. The existing authoritative studies on human gut microbiome composition and alcohol have sample sizes ranging between *N* = 13–66 [7,69,70]. Thus, the current study makes a robust addition to the literature at the intersection of clinical health and the human gut microbiome. Additionally, our analytical approach represents a methodological strength, as it is the first known application of factor analysis to study the underlying structure of the alpha diversity construct in relation to the human gut microbiome, thus enhancing the novelty and rigor of our analysis. Ultimately, the current investigation provides one of the first sources of empirical evidence of key alpha diversity sub-constructs (richness and evenness) within the human gut microbiome.

In addition to the methodological strengths discussed above, the study also has a number of limitations, and our results should be interpreted in the context of these considerations. First, though the sample size is moderate to large compared to previous published studies employing fecal samples from human clinical samples in the context of alcohol use, the current sample size is considered small in the context of factor analytic approaches, as discussed in Tabachnick & Fidell [53]. Authors note, however, that the sample size guidelines might be relaxed if the factor analysis solution include several high loading marker variables (< .8), as is the case in the current set of analyses. Additionally, the health-related variables of interest included in the current set of analyses (fruit and vegetable consumption, alcohol use behavior, depression symptomatology) are based on self-report data. Though each of the respective self-report measures that were used to operationalize our variables of interest are validated, psychometrically sound measures, future studies should consider ways of integrating objective measures of behavior.

Furthermore, the statistical analyses conducted in this study included several statistical tests. That is, 16 individual regression models were run in order to test the associations between health-related variables of interest (i.e., age, alcohol consumption, fruit and vegetable servings, BDI) and alpha diversity measures (i.e., richness composite, evenness composite, Faith pd, Strong). Type I error rate, which is the likelihood of getting a “false positive” result in the absence of a “true” effect, increases as the number of statistical tests increases. Given that 16 statistical tests were run, it is possible that some of the significant effects reported in the present investigation were the result of Type I error. Importantly, efforts were made to reduce the threat of Type I error. Specifically, we planned our statistical tests a priori, such that we selected the four health-related variables of interest and the two individual alpha diversity metrics prior to the start of data analysis. Finally, these data are cross-sectional in nature because these variables were measured at one single time point. Ultimately, our interest is in characterizing the causal relationships between manifestations of health and biological mechanisms. Therefore, future studies should examine microbiome composition over time, and how stability and/or changes in the human gut microbiome relate to changes in other biological and behavioral variables of interest over time.

### Directions for future research

While we feel that our study represents a meaningful contribution to the literature, our work also highlights the many important questions that were not addressed in the present investigation. Future investigations should examine the impact of moderating factors on gut microbiome alpha diversity. The study sample in the current investigation was 92.19% Caucasian and 49% female. Due to the sample size (n = 64 participants) we were not sufficiently powered to evaluate the impacts of race and gender, two potentially important moderating factors, on the gut microbiome. Therefore, it is not possible to determine if the results here extend to non-Caucasian individuals, and both male and female gender. Future studies with larger sample sizes and higher racial diversity should be conducted to address these issues. Additionally, future studies should seek to validate the factor structure found in the present investigation. The two-factor solution and the “richness” and “evenness” factor names are consistent with theory and with the interpretation of the EFA results in this study. It will be important to replicate these results using different samples. Towards these ends, researchers could run exploratory and/or confirmatory factor analyses using other datasets comprised of human fecal microbiome samples.

### Significance and concluding remarks

Based on the current investigation, results suggest that gut microbiome alpha diversity is a multi-dimensional construct, and thus researchers should consider operationalizing the gut microbiome alpha diversity with this complexity in mind. As demonstrated by the results of Aim 2 of the current investigation, different facets of gut microbiome complexity (i.e., richness vs. evenness) show different relationships with key variables of interest. Failing to account for the heterogeneity of the gut microbiome construct could mask true, significant associations between facets of microbiome alpha diversity and key variables related to human health. With this in mind, we are making two concrete methodological recommendations:

1. We suggest that the operationalization of microbiome alpha diversity should be comprised of separate “richness” and “evenness” measures. We make this suggestion because we found evidence that “richness” and “evenness” are two distinct sub-constructs of the broader alpha diversity construct. One method by which this can be accomplished is for researchers to create composite measures comprised of the individual alpha diversity indices that we used to construct our richness/evenness measures (i.e., richness = Fisher alpha, Faith pd, Shannon, Enspie, Menhinick; evenness = Strong, Simpson e, Heip e, Lladser pe). In order to further explore and justify this proposed factor structure, we also suggest that researchers test this factor structure by specifying CFA models.
2. When investigating the relationship between human health and behavioral variables of interest and microbiome diversity, researchers should use composite measures, comprised of multiple individual alpha diversity indices that measure the same alpha diversity sub-construct (i.e., richness, evenness). We make this suggestion because our results suggest that composite measures can provide information over and above individual alpha diversity metrics.

Based on our analysis, these recommendations should be considered for the following reasons: 1) composite measures are more robust and reliable compared to individual indices; 2) the richness and evenness sub-constructs are empirically derived based on analysis of a human sample; and 3) these composite measures demonstrate utility in predicting key health-related variables of interest. It is our hope that this investigation serves as a methodological guide for researchers across a host of disciplines.

## Supporting information

S1 Data (CSV)
